# Mesenchymal Stromal Cells Laden in Hydrogels for Osteoarthritis Cartilage Regeneration: A Systematic Review from In Vitro Studies to Clinical Applications

**DOI:** 10.3390/cells11243969

**Published:** 2022-12-08

**Authors:** Cristina Manferdini, Elena Gabusi, Yasmin Saleh, Enrico Lenzi, Giovanni D’Atri, Leonardo Ricotti, Gina Lisignoli

**Affiliations:** 1 IRCCS Istituto Ortopedico Rizzoli, Laboratorio di Immunoreumatologia e Rigenerazione Tissutale, 40136 Bologna, Italy; 2 The BioRobotics Institute, Scuola Superiore Sant’Anna, 56025 Pisa, Italy; 3 Department of Excellence in Robotics & AI, Scuola Superiore Sant’Anna, 56025 Pisa, Italy

**Keywords:** hydrogels, osteoarthritis, mesenchymal stromal cells, cartilage regeneration

## Abstract

This systematic review is focused on the main characteristics of the hydrogels used for embedding the mesenchymal stromal cells (MSCs) in in vitro/ex vivo studies, in vivo OA models and clinical trials for favoring cartilage regeneration in osteoarthritis (OA). PubMED and Embase databases were used to select the papers that were submitted to a public reference manager Rayyan Systematic Review Screening Software. A total of 42 studies were considered eligible: 25 articles concerned in vitro studies, 2 in vitro and ex vivo ones, 5 in vitro and in vivo ones, 8 in vivo ones and 2 clinical trials. Some in vitro studies evidenced a rheological characterization of the hydrogels and description of the crosslinking methods. Only 37.5% of the studies considered at the same time chondrogenic, fibrotic and hypertrophic markers. Ex vivo studies focused on hydrogel adhesion properties and the modification of MSC-laden hydrogels subjected to compression tests. In vivo studies evidenced the effect of cell-laden hydrogels in OA animal models or defined the chondrogenic potentiality of the cells in subcutaneous implantation models. Clinical studies confirmed the positive impact of these treatments on patients with OA. To speed the translation to the clinical use of cell-laden hydrogels, further studies on hydrogel characteristics, injection modalities, chemo-attractant properties and adhesion strength are needed.

## 1. Introduction

Osteoarthritis (OA) is a degenerative disease of the whole joint tissues that leads to a progressive loss of articular cartilage (AC), causing chronic pain and disability in the affected patients [1]. Different risk factors, such as age, gender, family history, obesity and traumatic injuries are involved in the pathogenesis of OA [2]. The evolution of OA is characterized by the production of catabolic mediators (interleukin (IL)1β, IL6 and tumor necrosis factor (TNF)α) responsible for the induction of inflammation and production of proteolytic enzymes (aggrecanases and matrix metalloproteinases (MMPs)) that contribute to producing damages in the joint tissues [3,4].

The AC protects bone surfaces within joints by providing a low-friction gliding surface for the articulation, supporting shock-absorption, distributing loads, reducing stresses on the subchondral bone and guaranteeing wear resistance [5]. The AC consists of chondrocytes immersed in an extracellular matrix (ECM) that is composed of 70% water and 30% organic components, such as aggrecan, collagen type 2, minor collagens (type 3–4, 9 and 11), proteoglycans, glycosaminoglycans and glycoproteins [6]. Lesions affecting AC have limited intrinsic capacity for self-regeneration [7]. Surgical, pharmacological or nonpharmacological treatments represent ways to only temporarily relieve the OA symptoms, making the regeneration of AC tissue an unmet clinical problem [8]. Recent progresses have demonstrated the potential of stem-cell-based therapies in the treatment of OA patients that are able to regenerate injured cartilage and at the same time attenuate on-going inflammation within the affected joint [9,10]. Thus, MSCs are stromal cells that can be isolated from different tissues such as bone marrow, adipose tissue, umbilical cord (UC) and blood, and they are capable of differentiating in cartilage, bone and adipose tissue [11,12]. They can produce growth factors, chemokines and cytokines and they possess the ability to migrate in the injured sites [13,14]. In vitro and in vivo studies have proved that MSCs with their secretome promote anti-inflammatory effects and contribute to the formation of cartilage tissue [15,16,17].

One of the most promising strategies in this regard is the use of MSCs combined with biomaterials [18,19]. Biomaterials must possess peculiar characteristics to be used for cartilage regeneration. Among the biomaterials, hydrogels are three-dimensional polymeric matrices that shows interesting features and that have potential for the treatment of cartilage defects [20]. In fact, hydrogels can be injectable or printable and can effectively embed viable cells without hampering their viability [20]. Hydrogel printability enables the creation of well-defined three-dimensional (3D) structures through 3D printing or other biofabrication technologies to mimic cartilage native tissues. Hydrogels are being widely employed as bioinks in 3D printing due to their tunable and injectable features [21,22]. A wide range of natural or synthetic biopolymers are available that can be combined to form hybrid hydrogels [20]. Moreover, gelation of hydrogel matrices can be achieved by physical or chemical crosslinking, creating structures with extraordinary water absorbing ability and a 3D network, such as the ECM [23]. The ECM of AC is rich mainly in polysaccharides and proteins, and hydrogels fabricated from these biopolymers have been widely studied. Recently, the importance of the properties of the hydrogel microenvironment that contribute to regulate stem cell chondrogenesis has been shown [24]. Among them, hydrogel mechanical properties such as stiffness and viscoelastic behavior have a role in guiding the cells to differentiation [25]. Stiffness is the capacity of a hydrogel to resist deformation in response to an applied force [26]. Viscoelasticity is the capacity of a hydrogel to exhibit both viscous and elastic behavior following the application of force [27]. Moreover, microstructural and spatial hydrogel properties such as porosity and anisotropy (hydrogel with well oriented structure) that create the architecture of the hydrogels also significantly influence the chondrogenic differentiation of MSCs [18,28,29]. Finally, it has been demonstrated that functionalized hydrogels with peptide or nanoparticles show positive effects on chondrogenic differentiation, with the ability to regulate cell activity and to show a tunable biodegradation profile [30,31,32,33].

Hydrogels can be excellent hosts for MSCs, and the therapeutic advantage of this strategy is to protect the cells injected into the defect (i.e., from shear forces and needle dimensions) and at the same time to favor their adhesion to the cartilage [20,25,26,34]. In fact, the hydrogels provide mechanical support, elasticity and stiffness and facilitate cell interactions with OA cartilage [5,25,26,35]. Finally, MSCs laden in hydrogel might contribute to ECM remodeling and maintenance of homeostasis [18,23,24]. Different narrative reviews have been published on this topic focusing on specific items [15,35,36]. This systematic review aims to use a defined search strategy that focuses on the main important characteristics of the hydrogels (material type, biofunctionalization, rheological properties, physical property and crosslinking methods), combined with different sources of MSCs and used in in vitro/ex vivo studies, in vivo OA models and clinical trials for favoring cartilage regeneration in OA.

## 2. Materials and Methods

### 2.1. Search Strategy

A systematic review was conducted in Pubmed and Embase databases from January 2011 to July 2022 considering the following keywords: adipose stem cells; mesenchymal stem cells; stromal cells; osteoarthritis; knee osteoarthritis; cartilage; chondrogenesis; cartilage regeneration and differentiation. The query box used for each study was “((mesenchymal stem cells OR stromal cells OR adipose stem cells) AND (hydrogel)) AND (cartilage OR chondrogenesis OR differentiation OR cartilage regeneration) AND (osteoarthritis OR knee osteoarthritis))”, filters “all fields”. Two independent researchers (Y.S. and E.G.) performed the screening process. Moreover, to overcome problems related to risk of bias assessment, we did not use a validated tool of assessment, but we scored the bias risk only if we found items that were not reported or unclearly reported. Finally, any disagreements were resolved by consensus with a third reviewer (C.M.).

### 2.2. Selection Process

**Figure 1:**
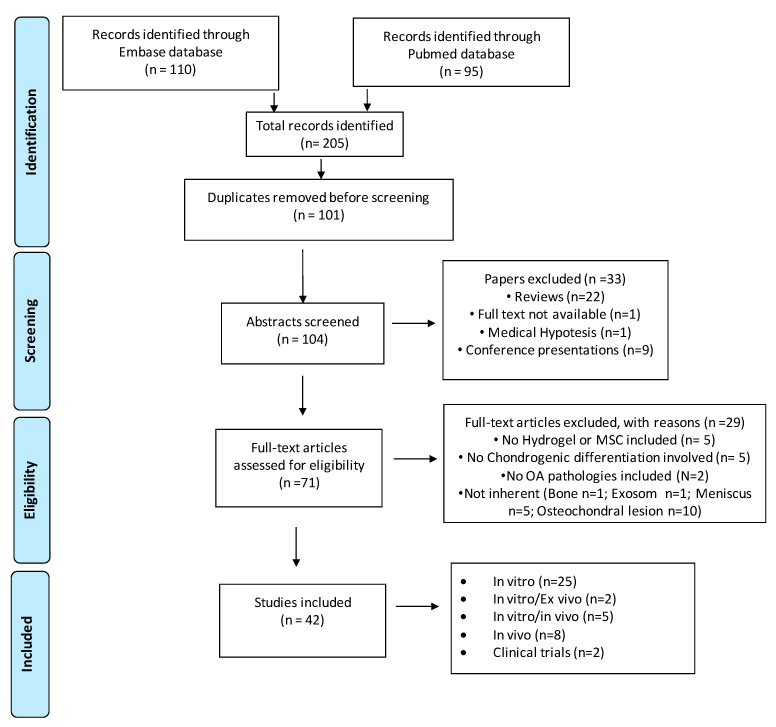
Search strategy according to Preferred Reporting Items for Systematic reviews and Meta-Analyses (PRISMA) guidelines and number of records found and included.

The selection of studies to be included was carried out following the PRISMA guidelines for systematic reviews. Starting from the results of Embase and Pubmed databases, a screening of the title and abstract and subsequently of the entire text of the article was performed using the free tool Rayyan online Software (https://www.rayyan.qcri.org, Qatar) (accessed on 30 July 2022). Articles written in other languages, abstracts, reviews, full texts not available, editorials or conference proceedings were excluded. The entire selection process is represented in the flow chart shown in Figure 1.

**Figure 2:**
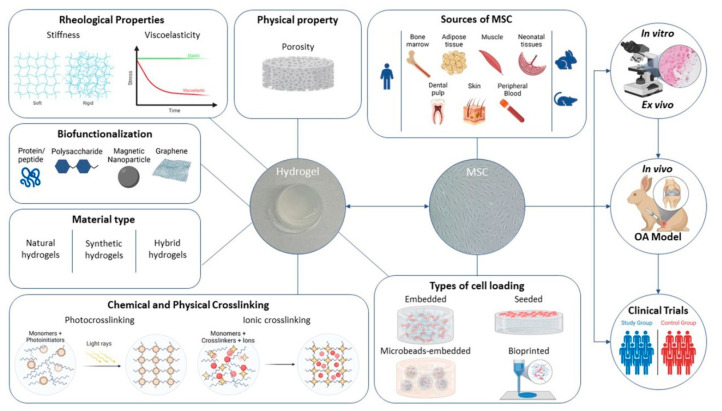
Major items analyzed in the selected studies, used to organize the review content.

As shown in Figure 2, all the information extracted from selected papers were grouped and the review was organized focusing on the following main items: hydrogel features (material type, biofunctionalization, rheological properties, physical property and crosslinking methods), MSC types and cell loading, experimental design (in vitro or ex vivo), in vivo OA models and clinical trials.

## 3. Results

### 3.1. Literature Search Strategy Results

The initial literature search retrieved 95 articles using PubMed and 110 articles using Embase by using the mentioned keywords. The selected references were submitted to a public reference manager Rayyan Systematic Review Screening Software (Qatar) to eliminate duplicate articles (*n* = 101). All the remaining abstracts (*n* = 104) were screened for excluding conference presentations (*n* = 9), reviews (*n* = 22), full text not available (*n* = 1) and medical hypothesis (*n* = 1) not matching with the inclusion criteria. A total of 71 articles were considered eligible. By evaluating the full text of all of the articles, a total of 29 articles were excluded because they focused on the following: regeneration of meniscus (*n* = 5), osteochondral lesions (*n* = 10), bone (*n* = 1), exosome treatment (*n* = 1), non-OA pathologies (*n* = 2), adipose or bone differentiation (*n* = 5) and hydrogels without MSCs (*n* = 5). As reported in Figure 1, a total of 42 studies were finally included in this review: 25 articles concerned in vitro studies, 2 in vitro and ex vivo results, 5 in vitro and in vivo results, 8 in vivo studies and 2 clinical trials.

### 3.2. In Vitro Studies

**Figure 3:**
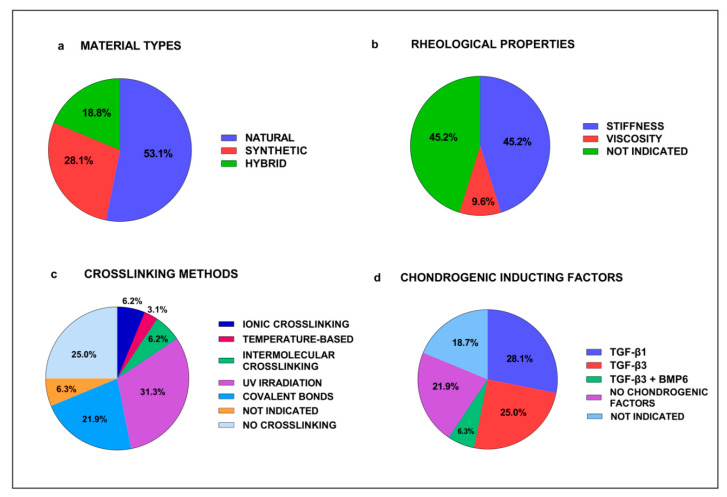
Major characteristics of *in vitro* studies: (**a**) material types; (**b**) rheological properties; (**c**) crosslinking methods; (**d**) chondrogenic inducting factors.

As reported in Table 1, we evidenced that in vitro studies were performed using different hydrogel types: 16 studies used natural hydrogels [12,37,38,39,40,41,42,43,44,45,46,47,48,49,50,51,52,53], 9 studies used synthetic hydrogels [37,54,55,56,57,58,59,60,61] and 7 studies used hybrid hydrogels [53,62,63,64,65,66,67] (Figure 3a). Among the hydrogels studied we found that 15 were non-functionalized [12,37,39,41,46,48,49,50,52,55,58,61,62,65,67] and 17 were functionalized [38,40,42,43,44,45,47,51,53,54,56,57,59,60,63,64,66] with peptide, growth factors or nanoparticles. Only Lu J et al. [38] studied a decellularized matrix hydrogel [46] that was functionalized with peptides. Interestingly, one study used microbeads [40] as a new method for analyzing chondrogenic differentiation and one study focused on a bioprinted hydrogel. The physical property of these hydrogels in term of porosity and the rheological properties of stiffness and viscosity were reported in 18 out of 32 studies. In particular, porosity was analyzed in 10 studies [12,45,46,51,54,56,58,60,65,66] that described the pore dimensions ranging from 1 to 100 µm [12,45,46,60,65,66] or the percentage of porosity ranging from 31% to 64% [51,54,56]. The stiffness was reported in 14 studies [42,45,46,48,49,50,51,53,59,60,61,62,65,66] that indicated the elastic modulus ranging from 0.12 kPa to 0.763 kPa or reported only a qualitative description. The viscosity was considered only in three studies [42,50,65] (Figure 3b).

Analyzing the mesenchymal stromal cells used we found that 20 studies used cells of human origin [37,40,41,42,43,44,45,46,47,48,49,52,53,54,56,58,59,60,62,68], two from rat [63,66], five from rabbit [38,51,57,61,65], two from goat [50,55], two from canine [39,64] and one from equine origin [12]. Only one study used MSCs derived from induced pluripotent stem cells (iPS) [64]. Among these in vitro studies, we evidenced that 27 of them [37,39,40,41,42,43,44,45,46,47,48,49,50,51,52,53,55,57,58,59,60,61,62,64,65,66,67] used cells embedded within the hydrogel matrix, while in 5 studies [12,38,54,56,63] the cells were seeded on the top of the hydrogels. Interestingly, analyzing the crosslinking methods used we evidenced that only 5 studies used physical crosslinking [42,51,58,66,67] (specifically, 2 used ionic crosslinking [42,58], 1 temperature-based methods [67] and 2 intermolecular crosslinking [51,66]) and 17 used chemical crosslinking [37,43,45,46,47,48,49,50,53,54,55,56,59,60,62,63,65], 10 with ultraviolet (UV) irradiations [37,43,47,48,49,50,54,55,56,59] and 7 with covalent bonds [45,46,53,60,62,63,65]). In two studies the method was not indicated [38,64] and in eight studies [12,39,40,41,44,52,57,61] hydrogels were not crosslinked (Figure 3c).

The chondrogenic differentiation was induced in vitro using the following growth factors: Transforming Growth Factor (TGF)β1 [12,39,40,50,52,55,59,63,65] or TGFβ3 [37,38,45,49,51,57,64,66], or TGFβ3 plus bone morphogenetic protein (BMP)6 [42,60]. In six studies [46,53,58,61,62,67] the factor used was not indicated and only seven studies [41,43,44,47,48,54,56] (Figure 3d) did not use growth factors.

Interestingly, in only one study [63] chondrogenic differentiation was enhanced by external inducing systems (i.e., pulsed electromagnetic field (PEMF)). Chondrogenic differentiation was analyzed from 7 to 56 days of culture, testing mainly the expression of typical chondrogenic markers such as collagen type 2 (COL2), SRY-box transcription factor 9 (SOX9), aggrecan (ACAN), glycosaminoglycans (GAG) or proteoglycans [12,37,38,39,40,44,46,49,51,52,54,55,56,57,58,62,63,64,66,67]. Some papers also considered the expression of fibrotic and hypertrophic markers, such as collagen type 1 (COL1), collagen type 10 (COL10) or MMP13 [41,42,43,45,47,48,50,53,59,60,61,65].

### 3.3. Ex Vivo Studies

Two in vitro studies previously described [49,60] also showed ex vivo results that are reported in Table 2. They focused on the integration and adhesive capacity [60] of the MSC-laden hydrogels to human OA cartilage and on their resistance to different strains delivered by a traumatic impact system [49]. Moreira-Teixeira et al. [60] analyzed the interaction and adhesion of a Dex-TA-based hydrogel to human OA cartilage with and without platelet lysate, evidencing by electron microscopy a close interaction with the cartilage specimen. He et al. [49] studied an engineered cartilage construct (GelMA hydrogel-BMSCs chondrogenically differentiated for 28 days) subjected to a traumatic impact system, evaluating the cell viability, cartilage gene modifications and the elastic modulus.

### 3.4. In Vivo Studies

**Figure 4:**
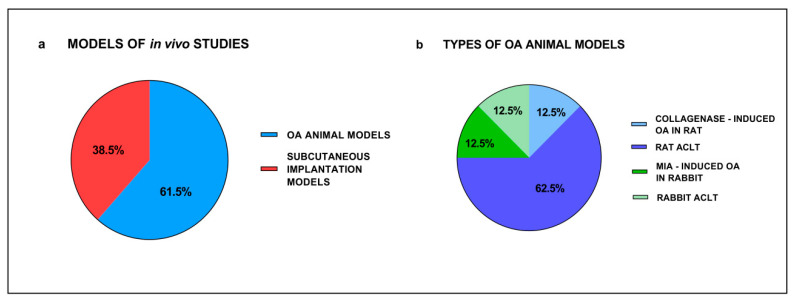
Types of in vivo studies (**a**) and types of OA animal models (**b**) in the selected articles.

As reported in Table 3, in vivo studies were performed in seven studies using natural hydrogels [47,69,70,71,72,73,74], in five synthetic hydrogels [57,59,61,75,76] and in one a hybrid hydrogel [66]. Interestingly, 7 out of 13 studies used non-functionalized [61,69,71,72,73,74,76] and 6 functionalized hydrogels [47,57,59,66,70,75]. The physical property of these hydrogels was reported for the porosity in two studies [66,70], the rheological features for stiffness in three studies [69,70,74] and for viscosity in one study [70]. Interestingly, only 3 studies used chemical crosslinking [47,59,72] and in 10 studies the hydrogels were not crosslinked [57,61,66,69,70,71,73,74,75,76]. In vivo studies were performed by applying them in eight OA animal models [61,66,69,70,71,73,74,76] and in five studies subcutaneous implantation models [47,57,59,72,74]. Among the studies that used OA models, six used rats, inducing OA by resection of ligaments and/or meniscus, one study used treatment with collagenase and two studies used rabbits, of whom one induced OA by ACL transection and one by monosodium iodoacetate (Figure 4).

All studies performed a knee joint injection of embedded cells one, two or three times. In two out of nine rat OA model studies, human umbilical cord blood (hUCB)-MSCs or mixed human embryonic stem cells with MSCs were used. All studies evidenced from 4 to 9 weeks an increase in chondrogenic markers (COL2, or ACAN or SOX9 or proteoglycan or GAG) [47,57,59,61,66,69,70,71,72,73,74,75,76] associated in some studies with a decrease in hypertrophic factors or inflammation or reduction in bone osteophytes or apoptosis [47,66,69,70,71,72,75,76].

In vivo subcutaneous dorsal implantation studies were performed in nude mice in four studies [47,57,59,72] and one in rats [74]. All MSCs used were of human origin and one of them overexpressed the long intergenic non-coding RNA regulator of reprogramming (Linc-ROR) [72]. All these studies evidenced an increase in chondrogenic markers from 2 to 8 weeks. Interestingly, Feng Q. et al. [47] and Feng L. et al. [72] reported a decrease in hypertrophic markers COL10 and MMP13. Only 4 out of 13 reported the dimension of the needles used for in vivo injection [69,70,75,76].

### 3.5. Clinical Trials

As reported in Table 4, two clinical trials were performed in OA patients with knee lesions [77,78]. Both studies treated the patients with multiple drill holes that were filled with non-crosslinked natural HA hydrogel combined with hUCB-MSCs [77,78]. In both studies the hydrogel characteristics in terms of rheological and physical properties were not reported. Different clinical (IKDC, WOMAC and VAS, KSS for pain, and arthroscopy), radiological (MRI) and histological parameters were analyzed to define the safety and/or the efficacy of the treatments.

## 4. Discussion

The regeneration of cartilage in OA disease remains an unmet problem that still requires the development of new approaches [8]. Hydrogels represent a promising tool, since they can easily embed viable cells such as MSCs or chondrocytes and can be easily injected in the defect area [20]. It has been shown that the hydrogels create a microenvironment that influences the cells’ characteristics, mainly due to their specific properties that have a positive or negative impact on the regulation of stem cell behavior [60]. Different papers have considered rheological and physical properties of the hydrogels, evidencing their direct role on cell chondrogenic differentiation. It has been shown that the material properties of hydrogels such as porosity, stiffness and viscoelasticity could modulate the cell characteristics; however, we have found that only a few papers have considered these important parameters. In fact, only in 7 [12,20,45,46,54,56,60] out of 42 papers the hydrogels’ porosity was analyzed by scanning electron microscope (SEM) analysis. Moreira Teixeira et al. [60] demonstrated that neither the culture medium nor the platelet lysate affected the pore size of a dextran-based hydrogel. It has been shown that pores ranging from 50–300 µm are suitable for modulating the cell shape, but also for cell adhesion, migration and diffusion of the nutrients and for stimulating the cell differentiation. The stiffness is an important hydrogel parameter, showing its capacity to resist deformation. This characteristic is fundamental for knee cartilage regeneration that is constantly under loading. However, we found that 17 papers [12,20,42,45,46,48,49,50,51,53,54,56,59,60,61,62,65,66,69,70,74] considered in their studies the stiffness. Interestingly, only Favi et al. [12] considered porosity (focusing on pore dimension, interconnectivity and fiber orientation) of the bacterial cellulose-based hydrogel. Viscoelasticity is the capacity of a hydrogel to exhibit both viscous and elastic behavior, and it has been shown that the increase in hydrogel stress relaxation promotes chondrogenesis. Only Yu et al. [70] considered the porosity, stiffness and viscoelasticity for developing an ECM-mimicking hydrogel. They demonstrated that a ThHA hydrogel functionalized with collagen type 1 (ThHA-Col) displayed the rheological properties that protect the cell survival and growth, having a stiffness close to the native microenvironment [70]. Moreover, they also evidenced that ThHA-Col exhibited shear-thinning properties that protect the cells during the injection.

Hydrogel functionalization is another important approach for driving and enhancing the MSCs to chondrogenic differentiation. The hydrogel functionalization was mainly based on the use of peptides/DNA [38,40,45,57,59,61,64,70,75] (i.e., RGD or collagen type 1 or 2 or glucosamine or pentosan polysulfate), nanoparticles [44,54,63] (i.e., drug-loaded or magnetic or graphene oxide) or soluble factors [44,47,53,56,60] (i.e., TGFβ, IGF-1 and platelet lysate), since each one of these factors have peculiar effects on chondrogenic cell boosting by increasing the main chondrogenic markers such as collagen type 2 and aggrecan. Among the clinically approved hydrogels as medical devices (Regenogel™ [52], JointRep™ Oligo Medic INC [47] and Caristem^®^ [77,78]), we evidence that no one was functionalized, and all were natural-based polymers (HA, fibrin and glucosamine). Moreover, to facilitate cell differentiation and nutrient transfer, recently the use of microbeads of hydrogels represents a new frontier as reported by Xing et al. [71].

It is well known that MSCs represent a promising cell tool for chondrogenic differentiation, and as reported in Table 1, Table 2, Table 3 and Table 4 human MSCs derived from bone marrow or adipose tissue or umbilical cord blood alone or combined with chondrocytes are the cell types mainly analyzed. However, MSCs from different animal sources (rabbit, canine, equine and rat) were also used to define their chondrogenic potentiality in hydrogels. MSCs have the potentiality to differentiate but at the same time are an important source of bioactive molecules that exert specific effects on chondrocyte proliferation and migration, as well as on immunomodulation. The embedding of MSCs in hydrogels represents an interesting approach for treating cartilage defects by injection. Hydrogel injection is a fundamental feature for the translation to the clinic, and only some in vivo studies [69,70,75,76] have reported the needle gauge (25 and 29 gauge), considering that it could be a parameter that could affect the cell viability by creating a shear stress that should be lower than 5 kPa, as previously reported [79]. Moreover, it is also important that injected hydrogel adhere well and remain stable in the defect area. Interestingly, Moreira Teixeira et al. [60] considered dextran-tyramine hydrogel adhesion to human OA cartilage, evidencing that the presence of tyramine residues contributed to the fixation of the hydrogel to collagen fibers or other matrix proteins of the cartilage. The authors also evidenced that the use of platelet lysate did not improve the cartilage adhesion but contributed to cell migration. The capacity of hydrogels to function as chemo-attractants for the cells is another important point for the clinical translation, since it contributes to assure a better integration of the hydrogel with the surrounding cartilage tissue. Finally, the mechanical properties of the engineered cartilage construct are another important point discussed by He et al. [49], using a loading system to mimic cartilage pressure.

Subcutaneous in vivo implantation studies [47,52,57,59,72,74] of MSC-laden hydrogels in nude mouse or rat models contributed only to defining the chondrogenic potentiality of the cells in a closer in vivo microenvironment but did not help for understanding OA disease effects.

In vivo studies based on the use of OA animal models [61,69,70,71,73,75,76] are fundamental for defining not only the chondrogenic potentiality of embedded MSCs in hydrogels but also for defining their effects on counteracting the inflammation that is a known feature in OA. Interestingly, Bhattacharjee et al. [69], Yu et al. [70] and Kim et al. [75], using collagenase-induced or ACL transection and medial meniscectomy or ACL and medial collateral transection in rats for inducing OA, demonstrated a significant reduction in inflammation and an increase in chondrogenic markers, such as collagen type 2 or GAG in treated animals. It is well known that the progression of OA disease is also characterized by the presence of osteophytes, and Yu et al. [70], Kim et al. [75] and Xing et al. [71] evidenced that MSCs embedded in hydrogel and injected in the knee were also effective in reducing osteophyte formation and restoring bone density.

The application of HA-based hydrogels embedded with umbilical-cord-blood-derived MSCs (Caristem^®^) is an approach already used in two clinical studies [77,78]. The authors applied hydrogel-laden MSCs to patients with OA knee lesions that were pre-treated with multiple drill holes. In one study [77], the follow-up was evaluated at 24 weeks and 7 years to define the efficacy and safety profile of the treatment. They evaluated different clinical scores (VAS, IKDC and MRI) and histological samples did not evidence undesired effects, but the number of included patients was limited to only three for each group. Regarding the other study [78], patients who underwent HTO for medial unicompartimental OA were preliminary treated with multiple drill holes and then divided into two groups, one treated with hydrogel-laden MSCs (32 patients) and one with bone marrow concentrate (42 patients). At 1 year follow-up, clinical and radiological outcomes were considered, and no differences were evidenced in term of WOMAC and KSS pain between the two groups. In a second look arthroscopy the ICRS grade was better in MSC-laden hydrogel treated patients, confirming that this treatment was the most efficient. The main limitations are the number of treated patients [78] and the use of MSC-laden hydrogel combined with multiple drill holes and compared with bone marrow concentrate, and not with MSCs alone as in the other study [77]. Finally, only one study considered the patients’ malalignment by performing HTO before the MSC-laden hydrogel treatment [78].

## 5. Conclusions

**Figure 5:**
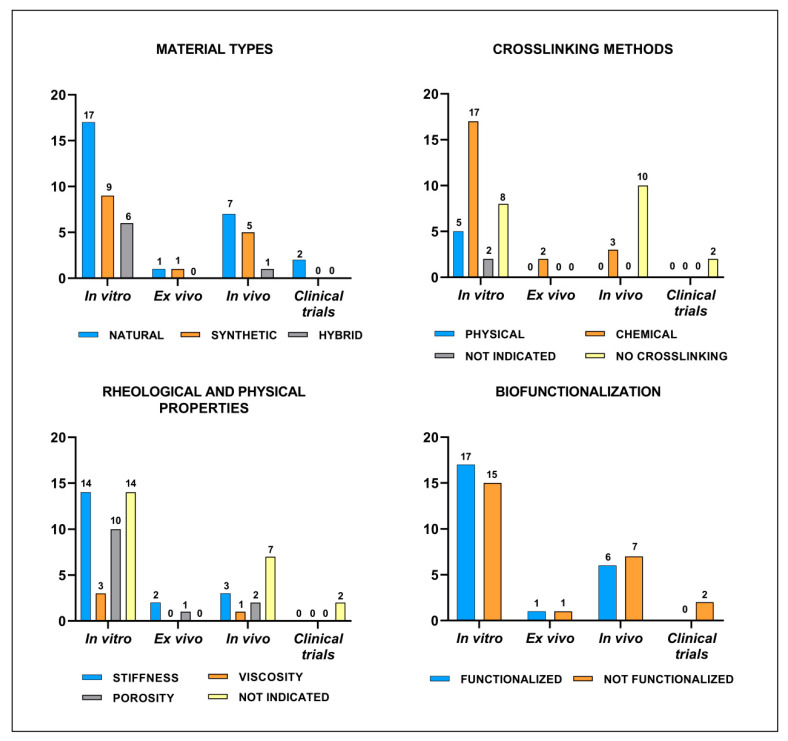
Summary of the main items evidenced in in vitro, ex vivo, in vivo and clinical trials. Histograms represent the number of articles included in the systematic review.

The need for new approaches to restore cartilage in OA disease is growing, and the use of MSC-laden hydrogel is a regeneration method that has been underlined in this systematic review, evidencing overall positive results that we summarized in Figure 5.

The positive in vitro results using different hydrogels and cell types were confirmed in in vivo OA animal models that well represent the progression of OA disease. Promising clinical trials confirmed the positive effect of these treatment on patients with OA. However, some aspects remain to be elucidated, mainly those focused on the material characteristics of the hydrogels used and on the cell type. Moreover, other aspects such as 3D bioprinting and crosslinking should be investigated in depth to provide better biocompatibility, as well as personalized and customized cartilage regeneration strategies. Additional hydrogel injection modalities, strength of adhesion to OA cartilage and the chemo-attractant role of the hydrogel need further studies that are fundamental to speeding the translation to the clinical use of this approach.

## Figures and Tables

**Table 1:** Summary of the main results of selected in vitro studies.

Hydrogel Type	Porosity (P), Stiffness (S) and Viscosity (V)	Functionalized with	Crosslinked	Cell Type and Loading	Chondrogenic Inducting Factors	Main Results	Reference
Fibrin/hyaluronan hydrogel (RegenoGel™) clinical approved as medical device	PSV—N.I.	No	No	Femoral-derived human bone marrow SCs (BMSCs) (embedded in hydrogel)	TGFβ1	↑of COL2, ACAN and GAG at day 28	Lolli et al. [52]
Aldehyde-modified hyaluronan (HAA) and hydrazide-modified polyvinyl alcohol (PVAH)	PV—N.I. S reported	No	Yes	Murine cell line W20-17 MSCs (embedded in hydrogel)	N.I.	↑of COL2, ACAN, GAG and proteoglycan at day 28	Aulin et al. [62]
Decellularized cartilage from porcine condyle	PSV—N.I.	Self-assembling peptides (RAD, PFS and RAD/PFS)	N.I.	Rabbit MSCs (seeded on hydrogel)	TGFβ3	↑of COL2, ACAN, SOX9 and COL1 at day 14 in hydrogel with RAD/PFS	Lu et al. [38]
40% polyethylene glycol (PEG)/60% polyethylene glycol diacrylate (PEGDA) printed hydrogel with a cold atmospheric plasma (CAP) treatment	P reported SV—N.I.	TGFβ1 loaded nanoparticles (NP)	Yes	Human MSCs (seeded on hydrogel)	No	↑of COL2, ACAN and SOX9 at day 21	S.J Lee et al. [54]
7% PEGDA-3% condroitin sulfate (CS)-methacrylate	PSV—N.I.	No	Yes	Human ASCs and osteoarthritic (OA) chondrocytes (C) mixed at different ratios (embedded in hydrogel)	TGFβ3	↑of COL2, ACAN, GAG and COL1 at day 21 in mixed culture (25C:75ASCs)	Lai et al. [37]
50% gelatin-50% beta-cyclodextrin	PSV—N.I.	Magnetic nanoparticles	Yes	Rat BMSCs (seeded on hydrogel)	TGFβ1	↑of COL2, ACAN and SOX9 at day 14 mainly after magnetic field treatment	Huang et al. [63]
10% PEGDA	PSV—N.I.	No	Yes	Goat BMSCs (embedded in hydrogel)	TGFβ1	↑of COL2 and GAG at day 21	Li et al. [55]
Polyglucosamine/glucosamine carbonate (PG/GC) (JointRep™ Oligo Medic INC.) clinical approved as medical device	PSV—N.I.	No	N.I.	Human ASCs (embedded in hydrogel)	N.I.	↑of COL2, proteoglycan and GAG at day 21	Pipino et al. [67]
Sulfated (S) methacrylate hyaluronic acid (MeHA)	PSV—N.I.	TGFβ1	Yes	Human MSCs (embedded in hydrogel)	TGFβ1	↑of COL2, ACAN and GAG ↓ of COL1 and COL10 at day 28 in low and high SMeHA	Feng Q. et al. [47]
Dextran-tyramine (Dex-TA)	PS reported V—N.I.	Incorporated human platelet lysate (hPL)	Yes	Human MSCs (embedded in hydrogel)	TGFβ3 + BMP-6	↑of COL2, GAG and proteoglycan and ↓of COL1 at day 21	Moreira Teixeira et al. [60]
Diacrylate PEG-DA (MWn = 700): PEG (MW = 300) (60% wt/wt)	P reported SV—N.I.	Nanocrystalline hydroxyapatite and TGFβ1	Yes	Human MSCs (seeded on hydrogel)	No	↑of COL2 and GAG at day 21	Castro et al. [56]
Silylated collagen	P reported SV—N.I.	Mimetic synthetic peptides	No	Human MSCs (embedded in hydrogel)	TGFβ3	↑of COL2, SOX9, ACAN and COL10 at day 21	Valot et al. [45]
Fibrin MeHA	PS reported V—N.I.	No	Yes	Human MSCs (embedded in hydrogel)	N.I.	↑of SOX9 (in presence of platelet lysate) at day 12	Snyder et al. [46]
DNA supramolecular	PV—N.I. S reported	No	No	Rabbit BMSCs (embedded in hydrogel)	N.I.	↑of COL2, SOX9 and ACAN and ↓ of COL1 and COL10 at day 14	Yan et al. [61]
Bacterial cellulose	P reported SV—N.I.	No	No	Equine MSCs (seeded on hydrogel)	TGFβ1	↑of proteoglycan and GAG at 7 and 14 days	Favi et al. [12]
Gelled platelet lysate	PSV—N.I.	No	No	Canine ASCs (embedded in hydrogel)	TGFβ1	↑of proteoglycan and GAG at day 28	Lima et al. [39]
Microbeads of agarose	PSV—N.I.	Different % of collagen type 2	No	Human MSCs (embedded in microbeads)	TGFβ1	↑of soluble GAG at day 21	Tiruvannamalai Annamalai et al. [40]
Self-assembled synthetic peptides	PSV—N.I.	Arginine-glycine-aspartate (RGD)	No	Rabbit ASCs infected with lentivirus-mature TGFβ3 (embedded in hydrogel)	TGFβ3	↑of COL2, ACAN and SOX9 at day 21	Zheng et al. [57]
PEG-hyaluronic acid (HA)	PSV—N.I.	Pentosan polysulfate	N.I.	Canine MSCs from induced pluripotent stem cells by inhibition the TGFβ/Activin signaling pathway (embedded in hydrogel)	TGFβ3	↑of proteoglycan and GAG at day 21	Whitworth et al. [64]
Methacrylated gelatin: HA (MeG:HA) ratios	PV—N.I. S reported	No	Yes	Human BMSCs (embedded in hydrogel)	TGFβ3	↑of COL2, SOX9 and ACAN and ↓ of COL10 at 56 days in 9:1 MeG:HA hydrogel ratio	Lin H et al. [48]
Collagen type 1	PSV—N.I.	No	No	Human BMSCs infected with adenoviral vector-(Ad)-SOX9, AdTGFβ1 and AdBMP2 (embedded in hydrogel)	No	↑of COL2, GAG and condroitin sulfate in all transduced hBMSCs at day 21; ↓of COL10 only in AdSOX9 transduced at day 21	Weißenberger et al. [41]
Thiolated gelatin (gelatin-SH)/PEGDA	PV—N.I. S reported	Insulin-Like Growth Factor (IGF)-1 cargo	Yes	Human ASCs (embedded in hydrogel)	N.I.	↑of COL2, ACAN, SOX9 and GAG and ↓ of COL1 at day 21	Cho et al. [53]
MeHA	PSV—N.I.	Microbeads of PEG/poly lactic acid-co-glycolic acid (PLGA) containing different concentrations of TGFβ3 or ghrelin	Yes	Human BMSCs (microsphere)	No	↑of COL2, ACAN and SOX9 and ↓of COL1 at day 10, in microbeads with 10 ng/mL TGFβ and 0.1 nM ghrelin	Lin J et al. [43]
Poly (N-isopropylacrylamide-co-acrylic acid (p(NIPAAm-AA) thermosensitive	SV—N.I. P reported	No	Yes	Immortalized human MSCs (UE7T-13) (embedded in hydrogel)	N.I.	↑of COL2, ACAN and SOX9 at day 28 and 35 and ↓of COL1 at day 42	Zhang J et al. [58]
Polyethylene glycol (PEG) PEGDA	PV—N.I. S reported	Glucosamine (10 mM)	Yes	Human BMSCs (embedded in hydrogel)	TGFβ1	↑of COL2, ACAN and SOX9 and ↓ of COL1, COL10 and MMP13 at days 21 and 42	Yao et al. [59]
Collagen type 1	PSV—N.I.	Graphene oxide adsorbed TGFβ3	No	Human BMSCs from OA patients (embedded in hydrogel)	No	↑of COL2, ACAN, SOX9 and GAG at day 28	Zhou et al. [44]
Thiolated gelatin crosslinked borate ester bond-based HA (HA-PBA)	PSV reported	No	Yes	Rabbit ASCs (embedded in hydrogel)	TGFβ1	↑of COL2, ACAN and SOX9 and ↓ of COL1, COL10 and MMP13 at days 14 and 28	Shi et al. [65]
Poloxamer 407 crosslinking of HA (PHA)	PS reported V—N.I.	Icariin	Yes	Rat BMSCs (embedded in hydrogel)	TGFβ3	↑of COL2, ACAN, SOX9, proteoglycan and Hypoxia Inducible Factor 1 Subunit α (HIF1α) at day 12 in icariin-embedded hydrogel	Zhu et al. [66]
VitroGel^®^	P—N.I. SV reported	RGD	Yes	Human ASCs (embedded in hydrogel)	TGFβ3 + BMP-6	↑of COL2, ACAN, SOX9, GAG and cartilage oligomeric matrix protein (COMP) and ↓of COL1 at day 28	Manferdini et al. [42]
Methacrylated gelatin (GelMA)	PV—N.I. S reported	No	Yes	Human BMSCs (embedded in hydrogel)	TGFβ3	↑of COL2, ACAN, SOX9 and proteoglycan at day 28	He et al. [49]
Methacrylated porcine decellularized cartilage ECM	P—N.I. SV reported	No	Yes	Goat MSCs (embedded in hydrogel)	TGFβ1	↑of COL2, GAG and ↓ of COL1 at day 21	Behan et al. [50]
Chondroitin sulfate (CS)	PS reported V—N.I.	Collagen type 1 and 2 blend	No	Rabbit BMSCs (embedded in hydrogel)	TGFβ3	↑of GAG and COL2 at day 28	Kilmer et al. [51]

N.I., not indicated; ↑, increased; ↓, decreased.

**Table 2:** Summary of the main results of selected ex vivo studies.

Hydrogel Type	Porosity (P), Stiffness (S) and Viscosity (V)	Functionalized with	Crosslinked	Ex Vivo Model	Cell Type and Loading	Chondrogenic Inducting Factors	Main Results	Reference
Dex-TA	**PS** reported **V**—N.I.	Incorporated hPL	Yes	Hydrogel adhesion to OA human cartilage	Human MSCs embedded in Dex-TA with and without PL chondrogenic induced for 8 days	TGFβ3 + BMP6	Dex-TA hydrogels/OA cartilage interface showed close interactions	Moreira Teixeira et al. [60]
GelMA	**S** reported **PV**—N.I.	No	Yes	Engineered cartilage construct subject to an impactor system	GelMA hydrogel-BMSCs chondrogenically induced for 28 days (engineered construct)	TGFβ3	Traumatic impact on engineered-construct-induced changes in cartilage genes and induction of chondrocyte catabolic genes	He et al. [49]

N.I., not indicated.

**Table 3:** Summary of the main results of selected in vivo studies.

Hydrogel Type	Porosity (P), Stiffness (S) and Viscosity (V)	Functionalized with	Crosslinked	Animal Model (Time to Develop OA or Time of Subcutaneous Implantation)	Cell type/ Hydrogel	Chondrogenic Inducting Factors	Gauge In Vivo Injection	Main Results	Reference
Sulfated (S) MeHA, two sulfate concentrations (low and high) tested	**PSV**—N.I.	TGFβ1	Yes	*Subcutaneous* implantation in nude mice (4 weeks) Rat anterior cruciate ligament transection (ACLT) and medial resection (4 weeks)	Human MSCs (embedded in hydrogel) preconditioned for 14 days in vitro before implantation	No	N.I.	↑COL2 and ACAN and ↓ COL10 and MMP13 at 4 weeks in high sulphate concentration	Feng Q et al. [47]
Decellularized human amnion	**PV**—N.I. **S** reported	No	No	*OA model*: rat collagenase-induced (1 week)	Knee joint injected with rat ASCs (embedded in hydrogel)	No	29 G	↓ of inflammatory factors, ↑of GAG at 4 weeks	Bhattacharjee et al. [69]
Thiolated-HA (ThHA)	**PSV** reported	Collagen type 1	No	*OA model*: rat ACLT and medial meniscectomy (4 weeks)	Knee joint injected with rat ASCs overexpressing TGFβ1 (embedded in hydrogel)	No	25 G	↓ of inflammatory factors and osteophytes, ↑of GAG and COL2 at 4 weeks	Yu et al. [70]
Gelatin-based 3D microgel	**PSV**—N.I.	No	No	*OA model*: rat ACLT (4 weeks)	Knee joint injected with human umbilical cord (UC)-MSCs (seeded in microgel)	N.I.	N.I.	↑of proteoglycan, COL1 and COL2; ↓of osteophytes both at 4 and 8 weeks	Xing et al. [71]
MeHA	**PSV**—N.I.	No	Yes	*Subcutaneous:* implantation in nude mice (2 weeks)	Human BMSCs overexpressing Linc-ROR (embedded in hydrogel) preconditioned for 2 weeks in vitro	N.I.	N.I.	↑of COL 2, SOX9 and ACAN, ↓ of MMP13 and COL10 at 2 weeks	Feng L et al. [72]
Self-assembled peptide (SAP)	**PSV**—N.I.	Neuropeptide (SP) different concentrations	No	*OA model*: rat ACL and medial collateral transections (2 weeks)	Knee joint injected with rat MSCs embedded in hydrogel	No	26 G	↓of inflammatory factors and bone density, ↑of SOX9 and COL 2 at 6 weeks; similar result with only SAP-SP	S.J Kim et al. [75]
1% Hyaluronan	**PSV**—N.I.	No	No	*OA model:* monosodium iodoacetate (MIA)-induced rabbit (2 weeks)	Knee joint injected three times (once every 3 weeks) with human embryonic stem cell-MSCs (embedded in hydrogel)	TGFβ	N.I.	↑of GAG and proteoglycan at 9 weeks	Zhang L et al. [73]
DNA supramolecular	**PV**—N.I. **S reported**	No	No	*OA model: rabbit ACLT and medial meniscectomy (MMx)*	Knee joint injected three times (once a week for 3 weeks) with rabbit MSCs embedded in hydrogel	N.I.	N.I.	↑of COL 2, GAG and proteoglycan at 6 weeks	Yan et al. [61]
Self-assembled synthetic peptides	**PSV**—N.I.	RGD	No	*Subcutaneous: implantation in nude mice (6 weeks)*	Rabbit ASCs preconditioned for 3 weeks in vitro before implantation (embedded in hydrogel)	TGF-β3	N.I.	↑of COL 2, ACAN, proteoglycan and GAG at 4 and 6 weeks	Zheng et al. [57]
Polyethylene glycol (PEG) PEGDA	**PV**—N.I. **S reported**	Glucosamine (10 mM)	*Yes*	*Subcutaneous: implantation in nude mice (8 weeks)*	Human BMSCs (embedded in hydrogel) preconditioned for 12 h in vitro before implantation	TGFβ1	N.I.	↑of COL2 and GAG at 8 weeks	Yao et al. [59]
Fibrinogen:trombin different ratios	**PV**—N.I. **S reported**	No	*No*	*Subcutaneous: * * implantation in rat * * (1 and 4 weeks)*	Human ASCs (embedded in hydrogel)	N.I.	N.I.	↑COL2 and GAG at 4 weeks in hydrogel with fibrinogen 30mg/mL: trombin 100IU/mL ratio	Kim J.S et al. [74]
SAP	**PSV**-N.I.	No	*No*	*OA model: rat ACLT, medial collateral transection and removal of medial meniscus (3 weeks)*	Knee joint injected rat MSCs (embedded in hydrogel)	No	26 G	↓ of inflammatory factors and apoptosis and ↑of COL2 2 at 6 weeks	Kim J.E et al. [76]
Poloxamer 407 crosslinking HA (PHA)	**PS reported** **V**—N.I.	Icariin	*Yes*	*OA model: rat destabilization of medial meniscus by medial collateral transection (2 weeks)*	Knee joint injected rat MSCs (embedded in hydrogel)	TGFβ3	N.I.	↓ of inflammatory factors and ↑of COL 2, SOX9, GAG and proteoglycan at 12 weeks	Zhu et al. [66]

N.I., not indicated; ↑, increased; ↓, decreased.

**Table 4:** Summary of the main results of selected clinical trials.

Hydrogel Type	Porosity (P), Stiffness (S) and Viscosity (V)	Functionalized with	Crosslinked	Study Design	Cells/ Hydrogel	Chondrogenic Inducting Factors	Patients Evaluated	Main Results	Reference
HA (Caristem^®^) clinically approved as medical device	**PSV**—N.I.	No	No	Phase I/II clinical trial in patients with moderate knee OA and painful full-thickness cartilage defects treated with multiple drill holes and divided in two groups: - Low-dose hUCB-MSCs embedded in HA (4 patients) - High-dose hUCB-MSCs embedded in HA (3 patients)	Human UCB-MSCs (Caristem^®^)	N.I.	At 24 weeks and 7 years by means of physical examination, VAS score for pain, IKDC, MRI and histological evaluations	The treatment had an acceptable efficacy and safety profile without undesired effects at 7 years	Park et al. [77]
HA (Caristem^®^) clinically approved as medical device	**PSV**—N.I.	No	No	High tibial osteotomy (HTO) for medial unicompartmental OA treated with multiple drill holes and divided in two groups: - bone marrow concentrate (42 patients) - hUCB-MSCs-HA (32 patients)	Human UCB-MSCs (Caristem^®^)	N.I.	At 1 year follow-up by means of IKDC, WOMAC and KSS pain and function scores	At 1 year no significant differences between the two groups; second-look arthroscopy after 1 year showed by ICRS grade a better regeneration of the cartilage in hUCB-MSCs-HA group	Lee N.H et al. [78]

N.I., not indicated.

## Data Availability

Not applicable.

## Abbreviations

3D	three-dimensional
AC	articular cartilage
ACAN	aggrecan
ACLT	anterior cruciate ligament transection
AdBMP2	adenoviral vector bone morphogenetic protein 2
AdTGFβ1	adenoviral vector Transforming Growth Factor Beta 1
AdSOX9	adenoviral vector SRY-Box transcription factor 9
ASCs	adipose-derived stromal cells
BMP6	bone morphogenetic protein 6
BMSCs	bone marrow stromal cells
CAP	cold atmospheric plasma
COL2	collagen type 2
COL1	collagen type 1
COL10	collagen type 10
COMP	cartilage oligomeric matrix protein
CS	chondroitin sulfate
Dex-TA	dextran-tyramine
ECM	extracellular matrix
GAG	glycosaminoglycans
GelMA	methacrylated gelatin
HA	hyaluronic acid
HA-PBA	borate ester bond-based hyaluronic acid
HAA	Aldehyde-modified hyluronan
HIF1α	Hypoxia Inducible Factor 1 Subunit Alpha
hPL	human platelet lysate
HTO	high tibial osteotomy
hUCB	human umbilical cord blood
ICRS	International Cartilage Regeneration Society
IGF1	Insulin-Like Growth Factor 1
IKDC	International Knee Documentation Committee Score
IL1β	interleukin 1 beta
IL6	interleukin 6
iPS	induced pluripotent stem cells
KSS	Knee Society Score
Linc-Ror	long intergenic non-coding RNA regulator of reprogramming
MeHA	methacrylate hyaluronic acid
MeG	methacrylated gelatin
MIA	monosodium iodoacetate
MMP13	matrix metalloproteinase 13
MMx	medial menisectomy
MRI	magnetic resonance imaging
MSCs	mesenchymal stromal cells
N.I.	not indicated
NP	nanoparticles
OA	osteoarthritis
P	porosity
PEG	polyethylene glycol
PEG/PLGA	polyethylene glycol-poly lactic acid-co-glycolic acid
PEGDA	polyethylene glycol diacrylate
PEMF	pulsed electromagnetic field
PFS	peptide sequence PFSSTKT
PG/GC	polyglucosamine/glucosamine carbonate
PHA	Poloxamer 407 crosslinking hyaluronic acid
PVAH	hydrazide-modified polyvinyl alcohol
RAD	GTP-binding protein RAD
RGD	arginine-glycine-aspartate
S	stiffness
SAP	self-assembled peptide
SEM	scanning electron microscope
SMeHA	sulfated methacrylate hyaluronic acid
SOX9	SRY-box transcription factor 9
TGFβ1	Transforming Growth Factor Beta 1
TGFβ3	Transforming Growth Factor Beta 3
ThHA	thiolated hyaluronic acid
ThHA-Col	thiolated hyaluronic acid hydrogel functionalized with collagen type 1
TNFα	tumor necrosis factor alpha
UV	ultraviolet
V	viscosity
VAS	visual analogue scale
WOMAC	Western Ontario and McMaster University Osteoarthritis Index
